# Highly effective sites and selectivity of nitrogen-doped graphene/CNT catalysts for CO_2_ electrochemical reduction†
†Electronic supplementary information (ESI) available: Limiting potential calculation; check for other possible active sites; activation barrier, DOS and curvature effect for some other structures; formation free energies of *COOH and *HCOOH intermediates for Edge-2gN structure under different curvature; formation energy of different N-doped graphene structures and unit cell size effect on intermediate formation energy. See DOI: 10.1039/c5sc03695j


**DOI:** 10.1039/c5sc03695j

**Published:** 2015-11-12

**Authors:** Guo-Liang Chai, Zheng-Xiao Guo

**Affiliations:** a Department of Chemistry , University College London , London WC1H 0AJ , UK . Email: z.x.guo@ucl.ac.uk

## Abstract

Metal-free catalysts, such as graphene/carbon nanostructures, are highly cost-effective to replace expensive noble metals for CO_2_ reduction if fundamental issues, such as active sites and selectivity, are clearly understood. Using both density functional theory (DFT) and *ab initio* molecular dynamic calculations, we show that the interplay of N-doping and curvature can effectively tune the activity and selectivity of graphene/carbon-nanotube (CNT) catalysts. The CO_2_ activation barrier can be optimized to 0.58 eV for graphitic-N doped graphene edges, compared with 1.3 eV in the un-doped counterpart. The graphene catalyst without curvature shows strong selectivity for CO/HCOOH production, whereas the (6, 0) CNT with a high degree of curvature is effective for both CH_3_OH and HCHO production. Curvature is also very influential to tune the overpotential for a given product, *e.g.* from 1.5 to 0.02 V for CO production and from 1.29 to 0.49 V for CH_3_OH production. Hence, the graphene/CNT nanostructures offer great scope and flexibility for effective tunning of catalyst efficiency and selectivity, as shown here for CO_2_ reduction.

## Abstract 2

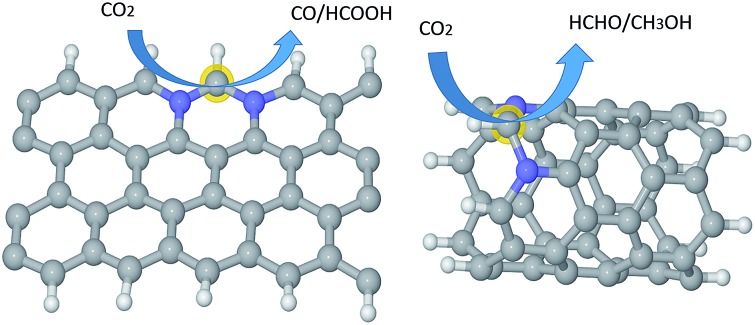
The selectivity of CO_2_ electrochemical reduction can be tuned for N-doped graphene/CNT catalysts after active sites are determined.

## Introduction

Excessive carbon dioxide (CO_2_) emission in the atmosphere leads to detrimental climate change. On the other hand, CO_2_ is a C1-building block for electrochemical or photochemical production of useful fuels and chemicals in industry, such as hydrocarbons, alcohols, organic acid and carbon monoxide.^1^–^5^ For instance, CH_4_ and CH_3_OH are desirable fuels for energy storage, and CO is widely used in chemical synthesis such as the Fischer–Tropsch and the Monsanto processes. However, challenges remain in CO_2_ conversion, such as poor efficiency and selectivity.^6^ For CO_2_ reduction, the low efficiency is mainly due to the difficulty of activation of the relatively stable molecule. The kinetic barrier for the first electron transfer to CO_2_, to form adsorbed CO_2_^–^, is rather high for most of the catalysts, usually above 0.70 eV as shown below, because this involves the bending of the linear and stable CO_2_ molecule. Moreover, different CO_2_ reduction products compete with not only each other but also with the electrochemical hydrogen evolution reaction (HER) in an aqueous solution, which leads to low selectivity. The catalysts should also have long durability under reducing conditions. Therefore, developing CO_2_ reduction catalysts that can overcome all these challenges is highly desirable.

In the past, the focus on CO_2_ reduction catalysts is mainly on metals (Au, Ag, Cu, Ru and Ni_5_Ga_3_ and so on),^7^–^11^ metal oxides^12^,^13^ and metal–organic complexes.^14^–^17^ Among those, Ag and Au show high selectivity for CO, Cu is the only metal shows selectivity for hydrocarbons, and Cu_2_O and RuO_2_ are favourable for methanol production.^12^,^13^ The products for metal–organic complexes catalysts are mainly CO, formic acid or oxalate, while formaldehyde (HCHO) is occasionally observed.^18^ However, the mechanism behind the selectivity is unclear due to the complex reaction processes and the rather short lifetime of relevant reaction intermediates. The active sites and reaction pathways are difficult to identify experimentally, though such an effort is highly significant for further improvement of product selectivity. Only recently, first principles simulations have been employed to elucidate the CO_2_ reduction mechanisms, which can identify the reaction intermediates at atomic scale.^19^–^24^

In a broader perspective, there is an increasing trend for the development of cost-effective metal-free catalysts, to substitute for noble metals. Currently such efforts are mainly focused on oxygen reduction reaction.^25^,^26^ The first experimentally investigated metal-free catalyst for CO_2_ reduction is N-doped carbons, which show a rather high overpotential for HER but low overpotential for CO_2_ to CO reduction.^27^ This study also claimed that the possible activation sites on the catalysts may be due to graphitic/quaternary N, which contradicts with another report that suggests the pyridinic N to be the active sites.^28^ Besides CO production, formate production was also observed for N-doped carbon catalysts in another study.^29^ Therefore, it is important to clarify the real active sites and understand the mechanisms for the selectivity. As mentioned above, CO_2_ reduction may compete with HER. Actually, it is well known that the *H intermediate is always more stable than both *COOH and *OCHO for almost all the catalysts developed for CO_2_ reduction to date,^30^–^32^ although a recent theoretical report predicting that the doping of lanthanide or actinide elements may reverse the situation.^33^ However, a chemical reaction is determined by both thermodynamics and kinetics. This is why high Faraday efficiency of CO_2_ reduction rather than HER was observed experimentally for N-doped carbon catalysts.^27^ For example, with the increase of pH value, it is more difficult to form the *H kinetically, while the activation of CO_2_ molecule is only slightly affected. Hence, we do not investigate HER systemically in the present study.

Moreover, tuning the selectivity for a wide range of useful products is highly meaningful in the development of this type of catalysts, which is currently lacking for metal-free carbon catalysts. There are only a few studies on metal–organic complexes, which show that the production of CO or formate can be tuned by means of different metallic or bimetallic centres.^34^,^35^ To gain such insight for graphene/CNT catalysts, a comprehensive mechanistic study is highly needed. To this end, we adopted both density functional theory (DFT) and *ab initio* molecular dynamic calculations to investigate the electrochemical reduction of CO_2_ on N-doped carbon catalysts, based on graphenes and carbon nanotubes (CNTs). As CO_2_ reduction performance is determined by both kinetic barriers and thermodynamic potentials, we first screened CO_2_ activation barriers for different structures to search for active sites. Then free energy variations between intermediates were calculated to clarify the selectivity of different products, such as CO, HCOOH, CH_3_OH, HCHO and CH_4_. Finally, we identified the very influential effect of curvature for tuning the limiting potentials in graphene catalysts for some practically important products (CO and CH_3_OH).

## Computational methods

**Fig. 1:**
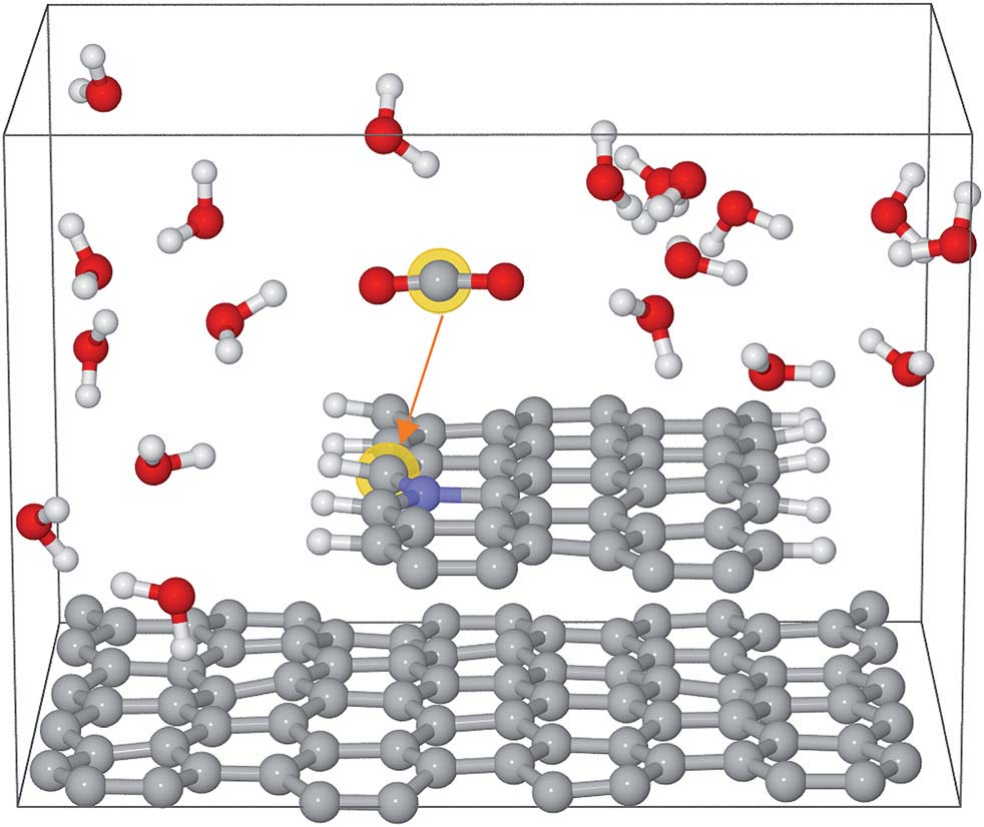
Simulation box for Edge-gN structure. The grey, blue, white and red spheres represent carbon, nitrogen, hydrogen and oxygen atoms, respectively.

The Car–Parrinello molecular dynamics simulations were performed at 300 K by means of the CPMD code with a time step of 4 a.u.^36^,^37^ The Blue Moon ensemble was employed to calculate the free energy barriers for CO_2_ activation.^38^ There are about 200 atoms in each simulation box, which contains a graphene bilayer structure (or graphene step edge), a certain number of water molecules and a CO_2_ molecule. An example of the simulation box is shown in Fig. 1. The sampling of the Brillouin zone was restricted to the Gamma point. The valence–core interaction is described by Troullier–Martins pseudopotentials (PP) for C, N, and O and von Barth–Car PP for H respectively.^39^,^40^ The GGA-HCTH exchange-correlation functional was adopted in a spin unrestricted scheme.^41^ The total energies were calculated by stationary DFT with PWSCF code in the Quantum ESPRESSO suite.^42^ Perdew–Burke–Ernzerhof (GGA-PBE) was used for exchange-correlation functional.^43^ Spin-polarization was adopted in all the calculations. The kinetic energy cutoffs for the wavefunction and the charge were set to be 35 Ry and 350 Ry, respectively. The single layer graphene or a CNT was employed for DFT calculations. The free energies are converted from calculated total energies by adding appropriate corrections to derive the limiting potentials as described in the ESI.†

## Results and discussion

### CO_2_ activation barriers

**Fig. 2:**
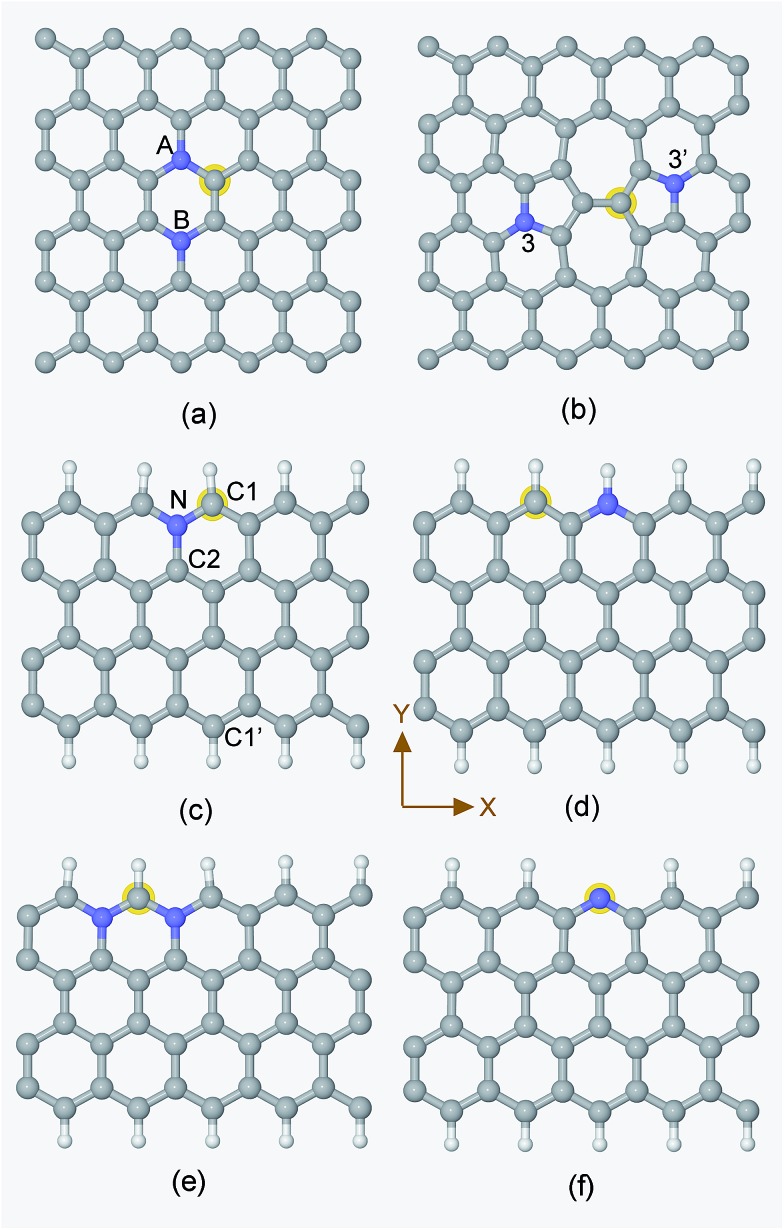
Unit cells for the periodic structures of (a) NN(AB), (b) SW-N3N3′, (c) Edge-gN, (d) Edge-pNH, (e) Edge-2gN and (f) Edge-pN, respectively. The G-N structure can be obtained from a NN(AB) structure by the substitution of the N atom in the B site by a C atom. The SW-N3 structure can be obtained from the SW-N3N3′ structure by the substitution of the N atom in the 3′ site by a C atom. The CO_2_ approaching site in each structure is labelled by a halo except for the Edge-pN at which the approaching site is N. The grey, blue and white spheres represent carbon, nitrogen and hydrogen atoms, respectively.

Generally speaking, the framework for N-doped carbon catalysts can be graphenes, CNTs, fullerenes or porous carbon structures *etc.* There are many different local N-doped configurations for each carbon framework, which makes the possible active sites complicated and unclear. So far, there is a lack of systemic investigation to determine the specific active sites for CO_2_ activation, although CO_2_ adsorption accompanied by the first electron transfer is usually the rate determining step for CO_2_ reduction, as mentioned above. The N-doped graphenes are employed here as a prototype of carbon catalysts to screen the local N-doped configurations and active sites for CO_2_ activation by means of *ab initio* molecular dynamic simulations. As shown in Fig. 2, the doped N can be in graphitic (gN), pyridinic (pN), or pyridinium (pNH) form in graphene based materials. Thus, the considered local configurations are gN doped perfect, Stone–Wales (SW) defect and zigzag edge graphenes, and pN/pNH doped zigzag edge graphenes, as shown in Fig. 2. For perfect and SW defect graphenes, both single-N and N-pair dopings were considered. As electrons need to be donated by electrode catalysts to CO_2_ molecule for CO_2_ activation and reduction, the C sites that possess high electronic density of states (DOS) just below the Fermi level are most likely candidates for the active sites and labelled by a yellow halo. Note here that the CO_2_ approaching site for the pN doped edge graphene is the N site rather than a C site. More details are discussed in ESI.†

**Fig. 3:**
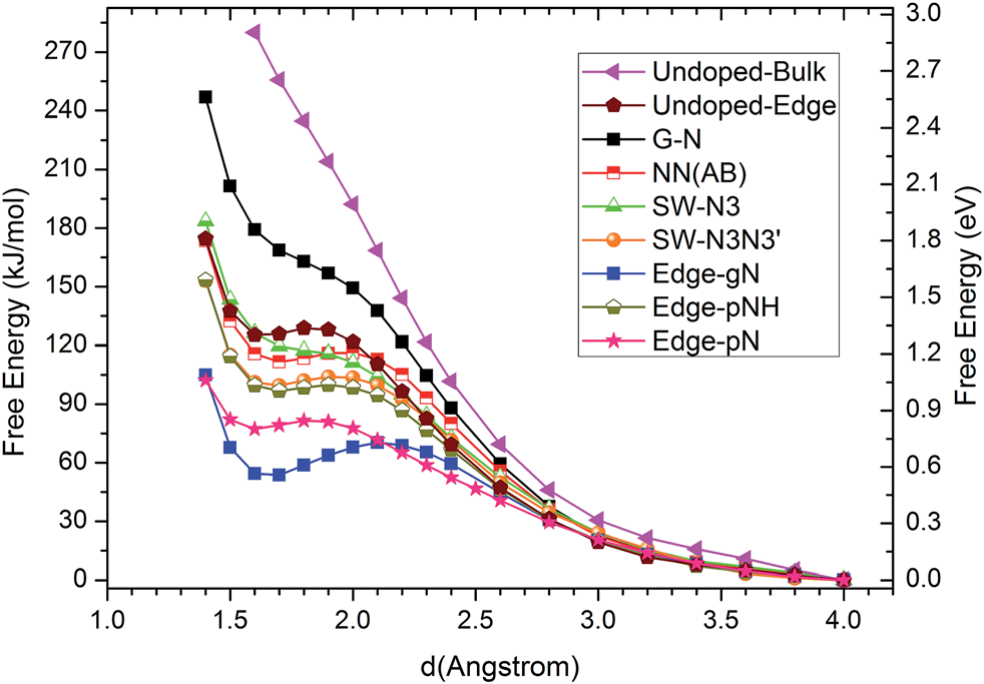
Free energy profiles of CO_2_ approaching C sites with large DOS just below the Fermi level in N doped graphenes for G-N, NN(AB), SW-N3, SW-N3N3′, Edge-gN, Edge-pN, Edge-pNH and un-doped bulk surface and edge structures. The approaching distance is that between C atom in CO_2_ and the candidate sites in catalysts. The free energy profile for O atom in CO_2_ approaching candidate sites are also checked in ESI.†

The corresponding CO_2_ activation free energy barriers for the considered candidate sites are shown in Fig. 3. A defect-free/undoped graphene surface does not possess a stable CO_2_ adsorption state and the CO_2_ approaching barrier is over 3.0 eV. The CO_2_ approaching barrier is reduced upon the doping of a single N atom to the graphene surface, but there is still no stable adsorbed state. When an N pair is doped in the A and B sites in an otherwise perfect graphene, the CO_2_ adsorption barrier continue to decrease to around 1.2 eV with a very shallow metastable adsorbed state. This barrier is still too high for CO_2_ reduction. If a SW defect is introduced to the graphene surface, the CO_2_ adsorption barrier is reduced further to about 1.1 eV, which is still relatively high for efficient CO_2_ reduction. For zigzag graphene edges, the un-doped edge shows a metastable CO_2_ adsorbed state with a barrier around 1.3 eV. The CO_2_ adsorption barriers for pNH, pN and gN doped zigzag edges are about 1.03, 0.84 and 0.72 eV, respectively. These barriers are very close to the experimental value of 0.71 ± 0.1 eV for CO_2_ reduction by a pyridine catalyst.^44^ The results indicate that both pN and gN can activate CO_2_, while the activation barrier for pN is larger than that for gN configurations. The adsorption barrier for gN and pN doped edges can be reduced by increasing the edge N concentration, as shown in Fig. S3† for Edge-2gN and Edge-2pN structures. The corresponding doped structures are shown in Fig. 2e and S4a,† respectively. Especially for gN doped edge (Edge-2gN), the barrier is reduced to 0.58 eV. The barrier for NN(AA) and gN doped fullerene structures are also checked: the barrier for NN(AA) is 1.01 eV, whereas there is no stable adsorbed state for the fullerene, as shown in Fig. S3.† Compared with O_2_ reduction, the activation of CO_2_ is clearly much more difficult.^45^ We only focus on gN doped structures subsequently, as those show low activation barriers.

**Fig. 4:**
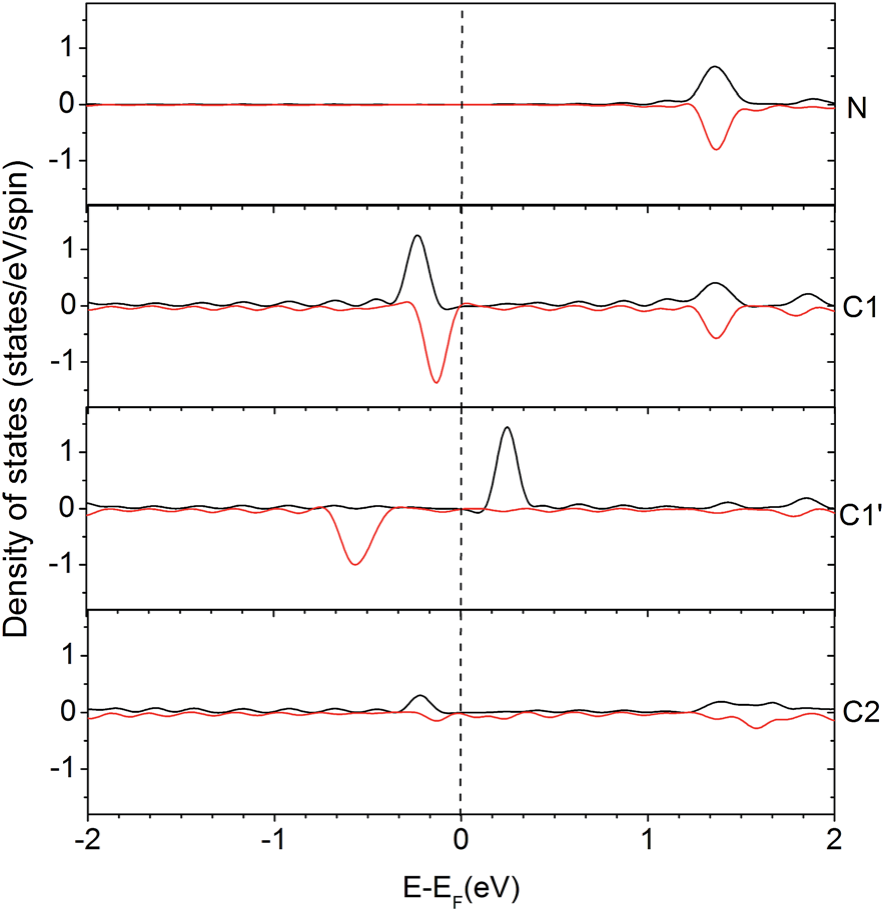
Density of states (DOS) for an edge-gN structure. The local density of states (LDOS) for N and some selected C atoms are presented (the two lines in each case represents the up- and down-spin states, respectively). The selected atoms are labelled in Edge-gN geometry structure in Fig. 2.

In order to understand the activity of the gN doped zigzag edge structure, the spin density of states (DOS) was calculated and shown in Fig. 4 (the corresponding geometry is shown in Fig. 2c). The ground state electronic configuration of a zigzag graphene edge is characterized by the ferromagnetic arrangement of spins along the edge and antiferromagnetic coupling of the spins at the opposite edge.^46^,^47^ Along the zigzag edge without N doping, the edge C1′ carbon atom shows unpaired but occupied electronic states just below the Fermi level and unoccupied electronic states just above the Fermi level, as shown in Fig. 4. For the gN doped zigzag edge, an electron is donated from gN to the unoccupied electronic states of C1 just above the Fermi level, which doubles its occupied electronic states just below the Fermi level and shifts those closer to the Fermi level, compared with those of C1′.^48^ The increased occupied electronic states just below the Fermi level of C1 readily facilitate electron transfer to a CO_2_ molecule. This is the main reason for the high activity of gN doped graphene with zigzag edges for CO_2_ reduction. For the C2 atom located near gN but not at the edge site, there is only a small amount of occupied electronic states just below the Fermi level, and it is almost zero for gN itself. Therefore, neither the C2 nor the gN is an efficient activity site. The free energy profiles for CO_2_ approaching gN sites are also checked and shown in the ESI† for comparison.

### Reaction pathways and selectivity

**Fig. 5:**
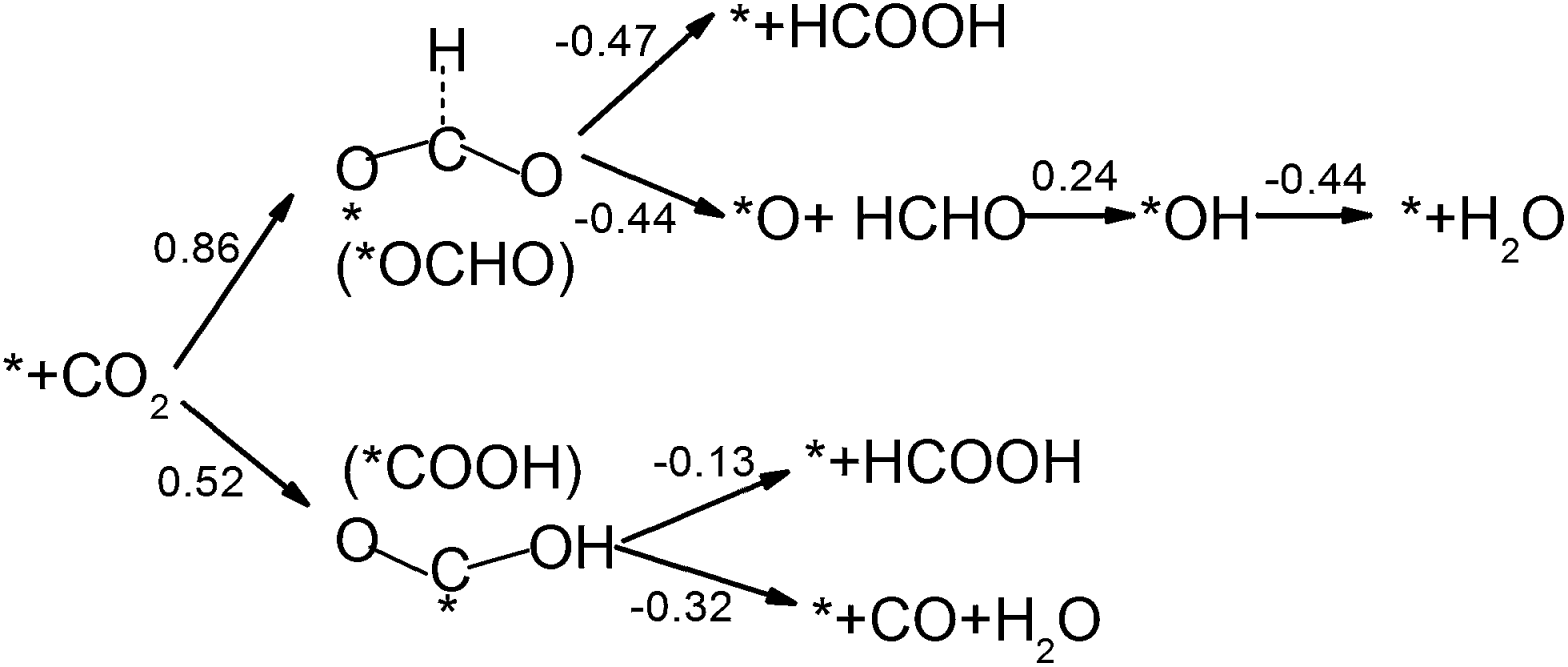
Free energy variations between intermediates for Edge-2gN graphene catalyst. (H^+^(aq) + e^–^) in each electron step is omitted for simplification. The “*” represents an active site.

**Fig. 6:**
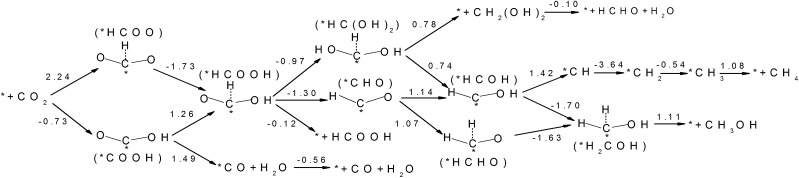
Free energy variations between intermediates for Edge-2gN (6, 0) CNT catalyst. (H^+^(aq) + e^–^) in each electron step is omitted for simplification.

CO_2_ activation is the first and usually the most difficult step for electrochemical reduction. After activation, different reaction pathways can lead to different final products. However, reaction intermediates and reaction pathways are difficult to identify experimentally, as mentioned above. Here, we calculated free energy variations for elementary steps of different reaction pathways to clarify the selectivity of CO_2_ reduction on the N-doped carbon catalysts. The Edge-2gN structure (the geometry is shown in Fig. 2(e)) was employed, as it shows the lowest activation barrier in this study. In order to tune the selectivity, the framework of graphene with zero curvature and a (6, 0) CNT with a large curvature were investigated, respectively. The results for selectivity of CO_2_ reduction are shown in Fig. 5 and 6.

For graphene catalysts, Fig. 5, there are four different reaction pathways during hydrogenation. The first hydrogen can be attached to the O site to form *COOH or the C site to form *OCHO intermediate. Note here, for hydrogen attached to the C site, the bond between the C atom and the catalyst surface is broken and then another bond is formed between the O and the surface, as shown in Fig. 5. Thermodynamically, the *COOH pathway (0.52 eV) is more favourable than the *OCHO pathway (0.86 eV). As there are no stable adsorbed states of *HCOOH and *CO on graphene surface for ongoing reduction, the *COOH intermediate can only form HCOOH or CO products by a two-electron reduction process. Here, the limiting potential for HCOOH and CO formation is identical under standard conditions (–0.52 V). For the *OCHO pathway, the second hydrogenation step also makes the intermediates desorb from the surface, and form HCOOH and HCHO, respectively. Therefore, for graphene catalyst with weak bonded intermediates, the selectivity is mainly for a two-electron reduction process and occasionally HCHO is observed. This observation agrees with the current experiments on carbon catalysts, which show that the main products are CO and HCOOH.^27^,^29^

Inspired by the curvature effect for O_2_ reduction to enhance bond strength,^45^ we introduced curvature to tune the selectivity for CO_2_ reduction on the N-doped carbon catalysts. The origin of the curvature effect mainly comes from the change of hybridization in the carbon electronic structure. For planar graphene without curvature, the C atom is in sp^2^ hybridization before adsorption and partial sp^3^ hybridization after adsorption of the intermediates. However, the well conjugated structure of graphene impedes the sp^2^ to sp^3^ conversion. Therefore, if partial sp^3^ hybridization exists before intermediate adsorption due to the curvature, the intermediate binding strength can be increased accordingly. Here, we use (6, 0) CNT to introduce a large degree of curvature to see how it changes the selectivity for CO_2_ reduction. From Fig. 6 we can see that the first hydrogenation step to the O site and the C site forms stable *COOH and *HCOO intermediates, respectively. This is different from the graphene surface that hydrogenation to C site would switch the O site bonding to the catalytic surface. However, the *COOH pathway (–0.73 eV) is much more favourable than the *HCOO pathway (2.24 eV) thermodynamically. Accordingly, we only need to consider the *COOH pathway. The second hydrogenation step can form stable *HCOOH and *CO intermediates, which is also different from graphene on which these two intermediates are desorbed. This suggests the possibility of ongoing reduction by a “more than two-electron” reduction process. Note here that now the *HCOOH is more favourable than *CO compared with that for graphene. For a two-electron reduction process on (6, 0) CNT, HCOOH formation now is more favourable than CO formation. For ongoing reduction, three final products are discussed for HCHO, CH_4_ and CH_3_OH. The rate determining step for HCHO and CH_3_OH formation is the same as for the formation of the *HCOOH intermediate, which makes the free energy increase by 1.26 eV. For CH_4_ production the rate determining step is for the formation of the *CH intermediate, with a free energy increase by 1.42 eV. Therefore, the formation of CH_4_ is more difficult than that of HCHO and CH_3_OH. The formation energy of *HCOOH intermediate is important for HCHO and CH_3_OH formation, which may be reduced by careful tuning of the curvature.

### Tuning limiting potentials by curvature effect

It can be seen that the selectivity of products of CO_2_ reduction can be tuned by curvature. Another question is that if the curvature is changed slowly would the potentials be tuned for the same product? Here, we tune the curvature effect slowly by reducing the lattice parameter along the X direction as shown in Fig. 2 for CO and CH_3_OH production as CO is the most simple reduction product and CH_3_OH is a very important liquid fuel for energy storage. A previous study also indicates that the activity for the same product can be tuned by different metal surfaces. For instance, the binding energy of the intermediate on the Pt(211) surface is too strong while that on the Au(211) surface is relatively weak for CO production.^49^ Fortunately, the intermediate binding energy is readily tuned by curvature in carbon materials catalysts. Generally speaking, there are two intermediates for CO_2_ to CO electrochemical reduction, *i.e.*, *COOH and *CO for strong bonding sites as shown in the case for (6, 0) CNT. However, for weak bonding sites in gN doped graphene catalysts, the *CO intermediate is unstable as discussed above. Here, we only focus on the cases without a stable CO adsorbed state (*CO) as the strongly bonded *CO intermediate may change the selectivity of products. The two elementary steps for CO_2_ reduction are as follows:

1 * + CO_2_(g) + (H^+^(aq.) + e^–^) → *COOH

2 *COOH + (H^+^(aq.) + e^–^) → * + CO(g) + H_2_O(l)

**Fig. 7:**
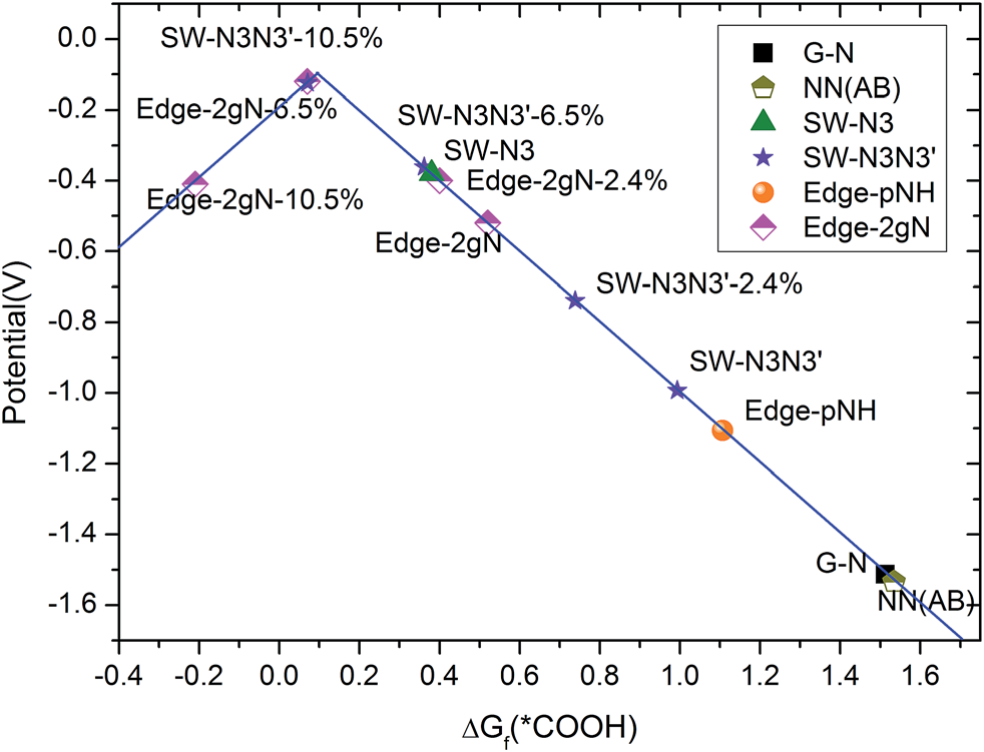
Calculated limiting potentials for CO_2_ to CO reduction. Curvature is added by reducing the lattice parameter along the *X* direction (shown in Fig. 2), which is presented by the percentage of lattice parameter reduced.

Limiting potentials can be derived from free energy variations. Here, the limiting potential is defined as the highest potential below which all the electrochemical steps are downhill in free energy and can be obtained from the free energy variation at the electrochemical reaction step. The calculated limiting potential can be compared with the experimental half-wave potential.^45^ Details for the calculation methods are presented in the ESI.† Overpotential is the absolute value of potential difference between a half-reaction's thermodynamically determined ideal potential and the onset potential at which the redox reaction is experimentally observed. As the onset potential is difficult to determine in computational chemistry, here we assume that the overpotential is the difference between the thermodynamically determined potential and the calculated limiting potential, although it would make the overpotential a little larger than 0–0.1 V. The calculated limiting potentials for the two-step two-electron CO_2_ reduction mechanism under standard conditions are shown in Fig. 7. The standard thermodynamically determined potential for CO_2_ to CO reduction is –0.1 V.^49^ It can be seen that the limiting potential for the gN doped perfect graphene is about –1.6 V (hence, the overpotential is 1.5 V). The limiting potential for the gN-pair doped SW defect is increased to –1.1 V. For the Edge-2gN structure, the limiting potential continues to improve to –0.52 V. However, the overpotential is still as large as 0.42 V. This means that the intermediate bond strength is still relatively weak for CO_2_ electrochemical reduction. By the introduction of curvature, the CO_2_ reduction limiting potentials can be tuned for different structures as shown in Fig. 7. For example, the overpotential for the Edge-2gN structure is tuned to 0.02 V, if the lattice parameter along the graphene edge is reduced by 6.5%. The overpotentials for the Edge-pN and the curved Edge-gN are shown in Table S2 in the ESI.† Note here that the Edge-pN goes through a three-step mechanism whereas the Edge-gN prefers a two-step mechanism. The results indicate that overpotentials for the Edge-pN cannot be reduced to zero due to the strongly adsorbed *CO intermediate. Similar to Edge-2gN, the overpotentials for the Edge-gN can also be tuned to nearly zero by curvature.

**Fig. 8:**
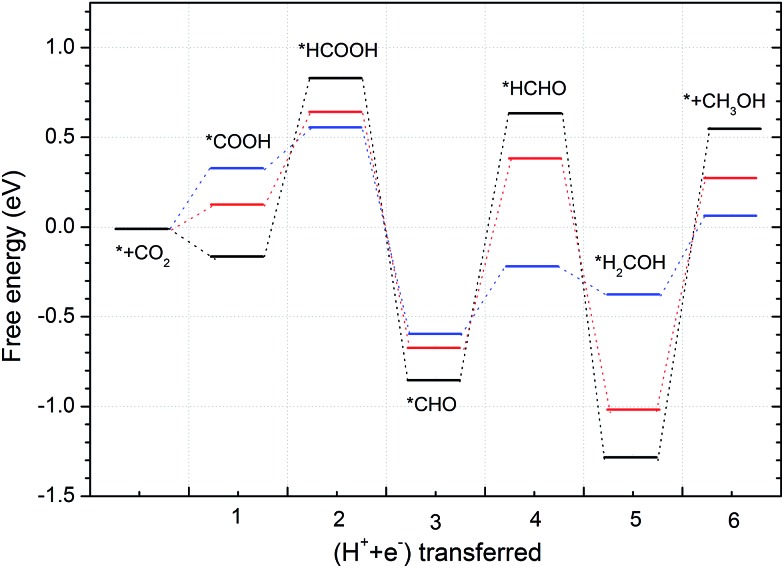
Free energy variations for CH_3_OH production. The black, red and blue lines correspond to the structures with the lattice parameter reduced for 10.5, 6.5 and 4.0%, respectively.

**Fig. 9:**
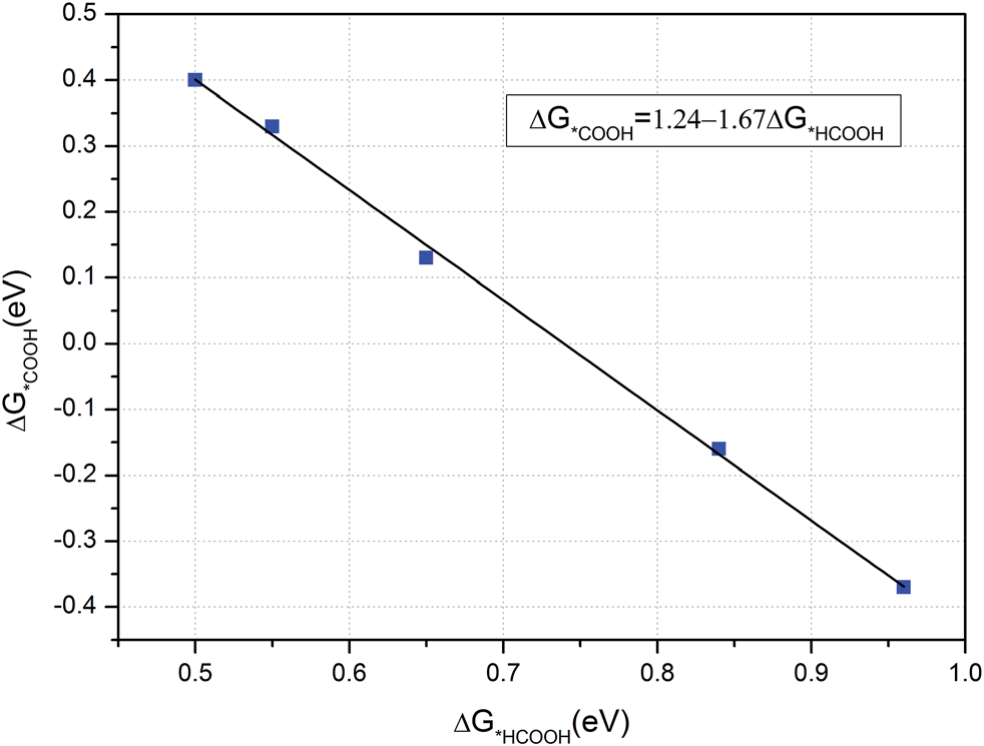
The relationship between formation free energy of *COOH and *HCOOH intermediates.

As CH_3_OH is an important liquid fuel, it is meaningful if the overpotential for CH_3_OH production is effectively reduced. Therefore, the curvature effect is also investigated for this product and the free energy variations are shown in Fig. 8. Note here that the *HCOOH intermediate is unstable on the flat graphene surface, but stable if the graphene is sufficiently curved. The most favourable reaction pathway for CH_3_OH production, Fig. 6, is selected for investigation. As shown in Fig. 8, the limiting potentials are tuned to be –0.84, –0.65 and –0.55 V when the lattice parameter is reduced by 10.5, 6.5 and 4.0%, respectively. It can also be seen that the energy limiting step is the formation of the *HCOOH intermediate from *COOH for all the different degrees of curvature studied. The formation energy of the *HCOOH intermediate decreases while that of *COOH increases with the decreasing of curvature, which suggests that the currently obtained limiting potential of –0.55 V can continue to be improved by careful tuning of curvature. Accordingly, the formation energies of *HCOOH and *COOH intermediates under different curvatures were studied and the results are summarized in Fig. 9. It can be seen that a linear relationship is well fitted between the formation energy of *HCOOH and *COOH intermediates. By means of this linear relationship, we can derive the optimum limiting potential for CH_3_OH production, under which the formation energy of *HCOOH and *COOH intermediates are identical, 0.46 eV. Therefore, the optimum limiting potential for CH_3_OH production is –0.46 V. As mentioned above, Cu_2_O and RuO_2_ catalysts can also be used for CH_3_OH production, but the Cu_2_O catalyst is unstable under reducing conditions. The RuO_2_ catalyst can be stable and generate CH_3_OH under a potential around 0.4 V *versus* RHE. However, its efficiency for CH_3_OH production is only 7.7%.^12^ The standard thermodynamically determined potential for CO_2_ to CH_3_OH reduction is 0.03 V. Hence, the minimum overpotential for CH_3_OH formation is 0.49 V, compared with the overpotential of 1.29 V on the (6, 0) CNT. Here, the N-doped carbon catalyst should be much more efficient – with a relatively low activation barrier and overpotential for CH_3_OH production. The value of formation energy of *HCOOH and *COOH intermediates are shown Table S3 in the ESI,† which indicates that the free energy of formation of *HCOOH intermediate is only 0.50 eV – if the lattice parameter is reduced by 2.4% under curvature. Hence, the optimum formation energy should be 0.46 eV, when the lattice parameter is reduced by less than 2.4%. Such a small reduction of lattice parameter can be readily realized, *e.g. via* graphene ripples or lattice constant mismatch between graphene and an underlying substrate.^50^ For example, “wrinkle” structures or nanobubbles of a width between 4 and 10 nm and a height around 0.3 to 2.0 nm are formed when graphene is grown on a platinum (111).^51^ The “wrinkle” structures or nanobubbles frequently appear near the edges of a graphene. If the “wrinkle” structures are approximated as triangles, the proportion of the curved area with reduced lattice parameters can be estimated to be from 0.18% (with width of 10 nm and height of 0.3 nm) to 29.3% (with width of 4 nm and height of 2 nm).

The formation energies for different N-doped configurations were also calculated using N_2_ gas as the nitrogen reference. The results are presented in Table S4 in ESI,† which agree with other first-principles studies.^52^ As shown in Table S4,† the NN(AA) structure shows the highest formation energy (2.04 eV). If the NN(AA) structure is at a graphene edge, (*i.e.* Edge-2gN structure), the formation energy is decreased to 1.03 eV, which is still relatively high compared with other structures. However, the results indicate that the formation energy along the edges is lower than in the bulk surface for the same local NN(AA) structure, which suggests the probability of realizing the Edge-2gN structure. Hence, here we propose two ways of realizing the Edge-2gN structure in practice. First, some molecular or polymeric precursors that already contain the NN(AA)-like local structure rather than N_2_ gas should be used as a nitrogen source. Second, some special synthesis methods should be employed to generate the local NN(AA) or Edge-2gN structure.^53^ Although ref. 53 focuses on the synthesis of NN(AA) in the bulk surface, the Edge-2gN structure may also be generated in other synthesis conditions, due to its lower formation energy. Alternatively, the Edge-2gN structure may also be obtained by cutting the NN(AA) structure along the zigzag direction to form zigzag edges.

The unit cell size effect were also checked by means of the formation free energies of *COOH intermediate for G-N and Edge-2gN structures with different unit cells. The results are shown in Table S5 in the ESI.† As shown in this table, the formation free energy errors (or unit cell size effect) are around 0.05 eV for both of the G-N and the Edge-2gN structures. The small errors mean that the final results will be slightly shifted but the conclusions remain the same.

## Conclusions

In summary, the activation barriers and selectivity of CO_2_ electrochemical reduction on N-doped carbon catalysts were investigated systemically by first principles simulations. The gN doped edge sites were identified to be the most effective for CO_2_ electrochemical reduction among a range of N doped sites in graphene/CNT catalysts, due to its special electronic edge states. The gN doped SW defect and pN doped edges are also possible active sites but the corresponding reaction barriers are higher than that for gN doped edge sites. The selectivity of CO_2_ electrochemical reduction has been investigated in two different structures: the graphene without curvature and the (6, 0) CNT with a significant degree of curvature. We found that the selectivity can be changed by the curvature effect. The graphene with weak bonding sites are favourable for CO/HCOOH formation, while the CNT with strong bonding sites are possible for HCHO and CH_3_OH formation. The limiting potentials can also be tuned for a given product under gradual change of curvature. For the same CO product, we find that a certain degree of curvature can improve the limiting potential for the Edge-2gN structure from –1.6 to –0.12 V, as shown in the volcano plot. The limiting potential for CH_3_OH production can also be tuned to around –0.46 V under curvature. The curvature can be realized experimentally, *e.g.* by means of naturally rippled graphenes, carbon nanotubes or porous structures. The study paves a solid foundation for future development of graphene/carbon catalysts for cost-effective and highly selective CO_2_ electrochemical reduction.

## Supplementary Material

Supplementary information

## References

[cit1] Angamuthu R., Byers P., Lutz M., Spek A. L., Bouwman E. (2010). Science.

[cit2] Rosen B. A., Salehi-Khojin A., Thorson M. R., Zhu W., Whipple D. T., Kenis P. J. A., Masel R. I. (2011). Science.

[cit3] Li H., Opgenorth P. H., Wernick D. G., Rogers S., Wu T. Y., Higashide W., Malati P., Huo Y. X., Cho K. M., Liao J. C. (2012). Science.

[cit4] Costentin C., Drouet S., Robert M., Savéant J. M. (2012). Science.

[cit5] Peterson A. A., Abild-Pedersen F., Studt F., Rossmeisl J., Norskov J. K. (2010). Energy Environ. Sci..

[cit6] Benson E. E., Kubiak C. P., Sathrum A. J., Smieja J. M. (2009). Chem. Soc. Rev..

[cit7] Mistry H., Reske R., Zeng Z., Zhao Z. J., Greeley J., Strasser P., Cuenya B. R. (2014). J. Am. Chem. Soc..

[cit8] Lu Q., Rosen J., Zhou Y., Hutchings G. S., Kimmel Y. C., Chen J. G., Jiao F. (2014). Nat. Commun..

[cit9] Manthiram K., Beberwyck B. J., Alivisatos A. P. (2014). J. Am. Chem. Soc..

[cit10] Matsubu J. C., Yang V. N., Christopher P. (2015). J. Am. Chem. Soc..

[cit11] Studt F., Sharafutdinov I., Abild-Pedersen F., Elkjær C. F., Hummelshøj J. S., Dahl S. R., Chorkendorff I., Nørskov J. K. (2014). Nat. Chem..

[cit12] Le M., Ren M., Zhang Z., Sprunger P. T., Kurtz R. L., Flake J. C. (2011). J. Electrochem. Soc..

[cit13] Karamad M., Hansen H. A., Rossmeisl J., Norskov J. K. (2015). ACS Catal..

[cit14] Saouma C. T., Lu C. C., Day M. W., Peters J. C. (2013). Chem. Sci..

[cit15] Bonin J., Robert M., Routier M. (2014). J. Am. Chem. Soc..

[cit16] Tornow C. E., Thorson M. R., Ma S., Gewirth A. A., Kenis P. J. A. (2012). J. Am. Chem. Soc..

[cit17] Medina-Ramos J., DiMeglio J. L., Rosenthal J. (2014). J. Am. Chem. Soc..

[cit18] Ramos-Sende J. A., Arana C. R., Hernandez L., Potts K. T., Keshevarz-K M., Abruna H. D. (1995). Inorg. Chem..

[cit19] Lim H. K., Shin H., Goddard W. A., Hwang Y. J., Min B. K., Kim H. (2014). J. Am. Chem. Soc..

[cit20] Keith J. A., Carter E. A. (2012). J. Am. Chem. Soc..

[cit21] Lim H. K., Shin H., Goddard W. A., Hwang Y. J., Min B. K., Kim H. (2014). J. Am. Chem. Soc..

[cit22] Lim C. H., Holder A. M., Hynes J. T., Musgrave C. B. (2014). J. Am. Chem. Soc..

[cit23] Schouten K. J. P., Kwon Y., van der Ham C. J. M., Qin Z., Koper M. T. M. (2011). Chem. Sci..

[cit24] Nie X., Esopi M. R., Janik M. J., Asthagiri A. (2013). Angew. Chem., Int. Ed..

[cit25] Ozaki J., Nozawa K., Yamada K., Uchiyama Y., Yoshimoto Y., Furuichi A., Yokoyama T., Oya A., Brown L. J., Cashion J. D. (2006). J. Appl. Electrochem..

[cit26] Gong K. P., Du F., Xia Z. H., Durstock M., Dai L. M. (2009). Science.

[cit27] Kumar B., Asadi M., Pisasale D., Sinha-Ray S., Rosen B. A., Haasch R., Abiade J., Yarin A. L., Salehi-Khojin A. (2013). Nat. Commun..

[cit28] Wu J., Yadav R. M., Liu M., Sharma P. P., Tiwary C. S., Ma L., Zou X., Zhou X. D., Yakobson B. I., Lou J., Ajayan P. M. (2015). ACS Nano.

[cit29] Zhang S., Kang P., Ubnoske S., Brennaman M. K., Song N., House R. L., Glass J. T., Meyer T. J. (2014). J. Am. Chem. Soc..

[cit30] Chen Z., Zhang X., Lu G. (2015). Chem. Sci..

[cit31] Tripkovic V., Vanin M., Karamad M., Bjorketun M. E., Jacobsen K. W., Thygesen K. S., Rossmeisl J. (2013). J. Phys. Chem. C.

[cit32] Karamad M., Tripkovic V., Rossmeisl J. (2014). ACS Catal..

[cit33] Cheng M. J., Kwon Y., Head-Gordon M., Bell A. T. (2015). J. Phys. Chem. C.

[cit34] Bagherzadeh S., Mankad N. P. (2015). J. Am. Chem. Soc..

[cit35] Chen L., Guo Z., Wei X. G., Gallenkamp C., Bonin J., Anxolabehere-Mallart E., Lau K. C., Lau T. C., Robert M. (2015). J. Am. Chem. Soc..

[cit36] Car R., Parrinello M. (1985). Phys. Rev. Lett..

[cit37] IBMCorp., CPMD 1990–2006, http://www.cpmd.org.

[cit38] Sprik M., Ciccotti G. (1998). J. Chem. Phys..

[cit39] Troullier N., Martins J. L. (1991). Phys. Rev. B: Condens. Matter.

[cit40] Sprik M., Hutter J., Parrinello M. (1996). J. Chem. Phys..

[cit41] Hamprecht F. A., Cohen A. J., Tozer D. J., Handy N. C. (1998). J. Chem. Phys..

[cit42] Giannozzi P. (2009). J. Phys.: Condens. Matter.

[cit43] Perdew J. P., Burke K., Ernzerhof M. (1996). Phys. Rev. Lett..

[cit44] Morris A. J., McGibbon R. T., Bocarsly A. B. (2011). ChemSusChem.

[cit45] Chai G. L., Hou Z., Shu D. J., Ikeda T., Terakura K. (2014). J. Am. Chem. Soc..

[cit46] Magda G. Z., Jin X., Hagymasi I., Vancso P., Osvath Z., Nemes-Incze P., Hwang C., Biro L. P., Tapaszto L. (2014). Nature.

[cit47] Lee H., Son Y. W., Park N., Han S., Yu J. (2005). Phys. Rev. B: Condens. Matter Mater. Phys..

[cit48] Huang S. F., Terakura K., Ozaki T., Ikeda T., Boero M., Oshima M., Ozaki J., Miyata S. (2009). Phys. Rev. B: Condens. Matter Mater. Phys..

[cit49] Hansen H. A., Varley J. B., Peterson A. A., Nørskov J. K. (2013). J. Phys. Chem. Lett..

[cit50] Guinea F. (2012). Solid State Commun..

[cit51] Levy N., Burke S. A., Meaker K. L., Panlasigui M., Zettl A., Guinea F., Castro Neto A. H., Crommie M. F. (2010). Science.

[cit52] Hou Z., Wang X., Ikeda T., Terakura K., Oshima M., Kakimoto M., Miyata S. (2012). Phys. Rev. B: Condens. Matter Mater. Phys..

[cit53] Lv R., Li Q., Botello-Mendez A. R., Hayashi T., Wang B., Berkdemir A., Hao Q., Elias A. L., Cruz-Silva R., Gutierrez H. R., Kim Y. A., Muramatsu H., Zhu J., Endo M., Terrones H., Charlier J. C., Pan M., Terrones M. (2012). Sci. Rep..

