# Supplementary material for: Highly effective sites and selectivity of nitrogen-doped graphene/CNT catalysts for CO2 electrochemical reduction
Source: Chem Sci. 2015 Nov 12;7(2):1268–75. doi: 10.1039/c5sc03695j (PMC5975832; doi:10.1039/c5sc03695j)
Supplement: Supplementary file 1 [file SC-007-C5SC03695J-s001.pdf]

Electronic Supplementary Material (ESI) for Chemical Science.
This journal is © The Royal Society of Chemistry 2015

Highly Effective Sites and Selectivity of Nitrogen-Doped Graphene / CNT Catalysts for CO_2 Electrochemical Reduction

Guo-Liang Chai, and Zhengxiao Guo*

Department of Chemistry, University College London, London WC1H 0AJ, United Kingdom

E-mail: z.x.guo@ucl.ac.uk

Limiting potential calculation.

In this study, we consider CO_2 reduction in acid solution under standard concentration. Therefore, the free energy of $(H^{+}+e^{-})$ equals to $1/2H_2$ for standard hydrogen electrode (SHE). The total energies of intermediates are calculated and converted to free energies by adding some corrections:

$$G = E_{Total} + E_{ZEP} + \int C_p dT - TS \qquad \text{(S1)}$$

where E_{Total} is the calculated total energy, E_{ZPE} is zero point energy, C_p is heat capacity, T is temperature, S is entropy. Here, the correction terms are present in Table S1. Most of the corrections are from previous literatures.[1–5] Additionally, a correction of -0.51 eV is applied to the CO molecule in the gas phase in order to correct systemic error for GGA-PBE functional as in a previous paper.[1] The solvation energy of -0.11 eV is added to each O atom in intermediates. For instance, the solvation energy of -0.11 eV and -0.22 eV is added for *OH and *COOH

intermediates, respectively. For two step formation of CO and HCOOH, the free energy variation of the second step is obtained by using reaction energy of the overall reaction to subtract the free energy of the first step. By applying this calculation method, the calculated limiting potential of the following overall half reaction of CO_2 reduction is -0.098 V vs SHE, which agrees well with the experimental value of -0.10 V.[1]

$CO_2(g)+2(H^+(aq)+e^-)\rightarrow CO(g)+H_2O(l)$ (S2)

Then, the limiting potential is defined as:

$U_L = Min_i[-\Delta G_i]/ne$ (S3)

where n is the number of electrons transferred for each electrochemical step (here n = 1 for the one-electron transfer step) and e is the elementary charge. The meaning of the *r.h.s.* of the above equation is to select the smallest $[-\Delta G_i]$ among a specific reaction pathway.

Check for other possible active sites.

The activation barriers for CO_2 approaching carbon sites with large DOS just below the Fermi level are shown in the main text. Here, the barriers for CO_2 approaching N sites are further checked for G-N and Edge-pNH structures, which show large barriers with no stable adsorbed states as presented in Figure S1. On the other hand, the C atom in CO_2 is selected to approach candidate active sites in the main text. Here, the O atom in CO_2 was selected to approach the candidate sites and the results are also shown in Figure S1. The results indicate that the barrier is much larger than that for C

atom approaching a given candidate site. This is because the C atom in CO_2 dominates the lowest unoccupied molecular orbital (LUMO) of CO_2 as shown in Figure S2.

Activation barrier, DOS and curvature effect for some other structures.

Other possible active sites around NN(AA), fullerene structures are also checked and the results are presented in Figure S3. The NN(AA) structure shows large activation barrier of 1.01 eV, while gN doped fullerene shows no stable adsorbed state. The adsorption barrier for increased edge N concentration were calculated for Edge-2gN and Edge-2pN structures as shown in Figure S3. The corresponding geometry structures are shown in Figure S4. The DOS of Edge-pN and Edge-pNH structures are shown in Figure S5 and Figure S6. The corresponding geometry structures are shown in Figure S4.

For Edge-pNH structure, the DOS just below the Fermi level for edge C2 and N sites are similar and larger than that of C1 site. The DOS of C2 is a little closer to Fermi level than that of N site. Therefore, the C2 site is selected as candidate site for Edge-pNH structure. For Edge-pN structure, both N and C2 sites have large DOS just below the Fermi level as shown in Figure 6. As there is a dispute for pN or gN as active sites, here we select the pN site as candidate site to compare with gN doped cases.[6,7]

The curvature effect for CO_2 to CO reduction on Edge-gN and Edge-pN

structures are also checked and presented in Table S2. As shown in this table, the overpotenital for Edge-gN structure can be tuned to nearly zero under certain curvature while it is not for Edge-pN structure. This mainly because the *CO intermediate is stable on Edge-pN structure, and the last CO desorption step is a non-electrochemical step whose energy is lost.

Formation energy of different N-doped graphene structures and unit cell size effect on intermediate formation energy.

The formation energies for different N-doped graphene structures are calculated by using N_2 gas as reference for nitrogen. The results are presented in Table S4. The formation energies are given by:

$$\Delta E_f(i\mathrm{N}) = E(i\mathrm{N}) - E_0 + iE(\mathrm{C}) - (i/2)*E(\mathrm{N_2}) \qquad \text{(S4)}$$

where i is the number of doped N atoms, $\Delta E_f(i\mathrm{N})$ is the formation energy, $E(i\mathrm{N})$ is the total energy of N-doped graphene. E_0 is the total energy of undoped graphene, $E(\mathrm{C})$ is the total energy per atom of perfect graphene. $E(\mathrm{N_2})$ is the total energy of an isolated N_2 molecule.

The formation free energies of *COOH intermediate are calculated for G-N and Edge-2gN structures with different unit cells to check the unit cell size effect. The results are shown in Table S5, which indicate that the unit cell size effect errors on formation are around 0.05 eV. The small errors would not change the conclusions but the final results of calculated potentials would be slightly shifted.

References

1 H. A. Hansen, J. B. Varley, A. A. Peterson and J. K. Nørskov, *J. Phys. Chem. Lett.,* 2013, **4**, 388-392.

2 M. Karamad, H. A. Hansen, J. Rossmeisl and J. K. Nørskov, *ACS Catal.,* 2015, **5**, 4075-4081.

3 P. Hirunsit, *J. Phys. Chem. C,* 2013, **117**, 8262-8268.

4 L. C. Grabow and M. Mavrikakis, *ACS Catal.,* 2011, **1**, 365-384.

5 A. A. Peterson, F. Abild-Pedersen, F. Studt, J. Rossmeisl and J. K. Nørskov, *Energy Environ. Sci.,* 2010, **3**, 1311-1315.

6 J. Wu, R. M. Yadav, M. Liu, P. P. Sharma, C. S. Tiwary, L. Ma, X. Zou, X. D. Zhou, B. I. Yakobson, J. Lou and P. M. Ajayan, *ACS Nano,* 2015, **9**, 5364-5371.

7 B. Kumar, M. Asadi, D. Pisasale, S. Sinha-Ray, B. A. Rosen, R. Haasch, J. Abiade, A. L. Yarin and A. Salehi-Khojin, *Nat. Commun.,* 2013, **4**, 2819.

Table S1: Corrections including zero point energy, heat capacity and entropy for converting calculated total energy to free energy (Unit is eV).

Species	ZEP	TS	∫ CpdT
*CO	0.22	0.08	0.05
*COOH	0.63	0.17	0.09
*HCOO	0.44	0.10	0.057
*HCOOH	0.82	0.085	0.049
*CHO	0.48	0.18	0.07
*$HC(OH)_2$	1.19	0.13	0.071
*HCOH	0.76	0.068	0.11
*HCHO	0.80	0.018	0.012
*H_2COH	1.06	0.22	0.11
*OCHO	0.63	0.20	0.10
*CH	0.35	0.039	0.028
*CH_2	0.59	0.075	0.049
*CH_3	0.90	0.096	0.06
*O	0.08	0.03	0.02
*OH	0.40	0.04	0.03
CH_3OH	1.35	0.79	0.11
CO_2	0.31	0.65	0.10
CO	0.14	0.67	0.09
H_2O	0.58	0.65	0.10
CH_4	1.20	0.60	0.10
HCHO	0.71	1.04	0.21
H_2	0.27	0.42	0.09
$CH_2(OH)_2$	1.51	1.11	0.20

Table S2: Free energy variation and overpotential for Edge-pN and Edge-gN structures. Curvature is added by reducing the lattice parameter along X direction, which is presented by the percentage of lattice parameter reduced.

Structures	ΔG_1	ΔG_2	ΔG_3	Overpotential (V)
Edge-pN-0%	0.18	1.09	-1.08	0.99
Edge-pN-2.4%	0.10	1.38	-1.29	1.28
Edge-pN-6.5%	-0.28	1.34	-0.86	1.24
Edge-pN-10.5%	-0.49	1.38	-0.70	1.28
Edge-gN-0%	0.59	-0.39	–	0.49
Edge-gN-2.4%	0.51	-0.31	–	0.41
Edge-gN-6.5%	0.19	0.01	–	0.09
Edge-gN-7.3%	0.11	0.09	–	0.01
Edge-gN-10.5%	-0.15	0.35	–	0.25

Table S3: Formation free energies of *COOH and *HCOOH intermediates for Edge-2gN structure under different curvature (Unit is eV).

Structures	ΔG_{*COOH}	ΔG_{*HCOOH}
Edge-2gN-2.4%	0.40	0.50
Edge-2gN-4.0%	0.33	0.55
Edge-2gN-6.5%	0.13	0.65
Edge-2gN-10.5%	-0.16	0.84
Edge-2gN-14.6%	-0.37	0.96

Table S4: Formation energies of different N-doped graphene structures (Unit is eV).

Structures	ΔE_f
G-N	0.82
NN(AA)	2.04
NN(AB)	1.68
SW-N3	0.03
SW-N3N3’	-0.26
Edge-pN	-2.83
Edge-pNH	-1.01
Edge-gN	0.11
Edge-2gN	1.03

Table S5: Formation free energies of *COOH intermediates for G-N and Edge-2gN structures with different unit cell size (Unit is eV). The supercell of 5×6 and 5×3 are employed in the main text.

Structures	Size	ΔE_f
G-N	5×6	1.51
	7×8	1.55
Edge-2gN	5×3	0.52
	5×7	0.57
	9×3	0.58

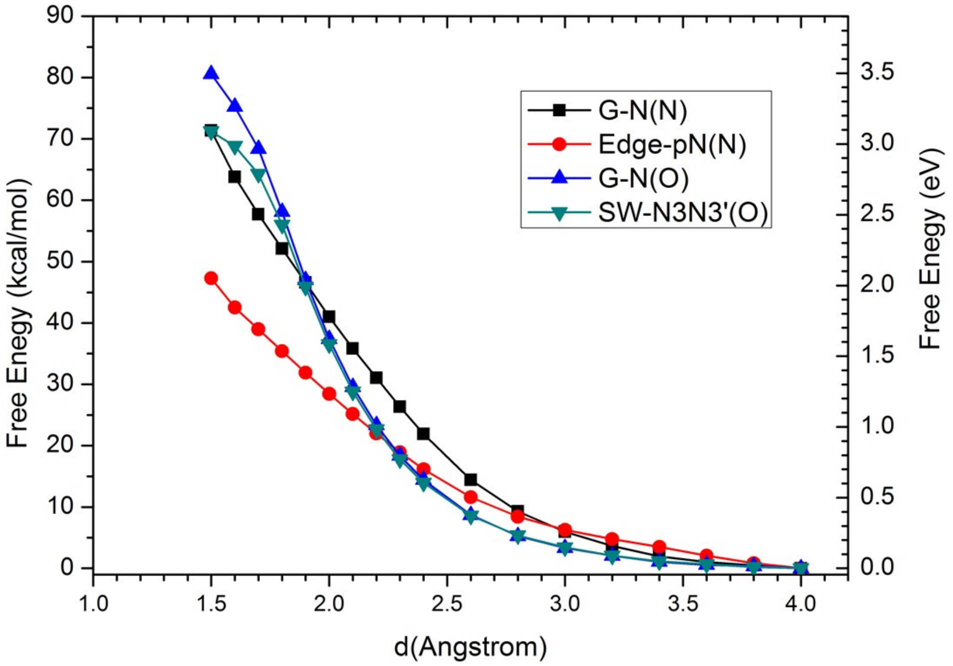

Figure S1: G-N(N) and Edge-pNH(N) are free energy profiles for C atom in CO_2 approaching N sites for G-N and Edge-pNH structures. The G-N(O) and SW-N3N3' (O) are free energy profiles for O atom in CO_2 approaching C sites with large DOS just below the Fermi level for G-N and SW-N3N3' structures.

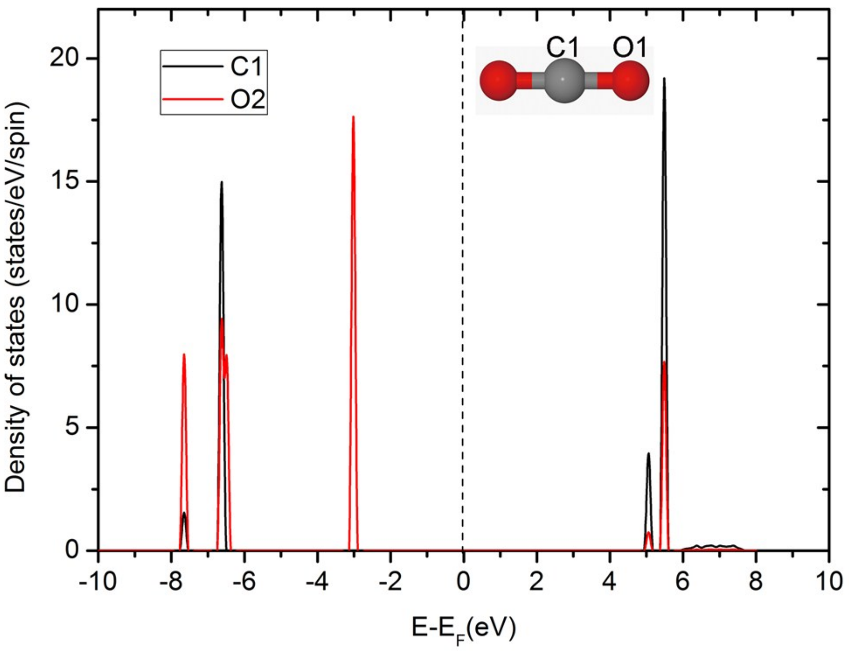

Figure S2: Density of states (DOSs) for a CO_2 molecule.

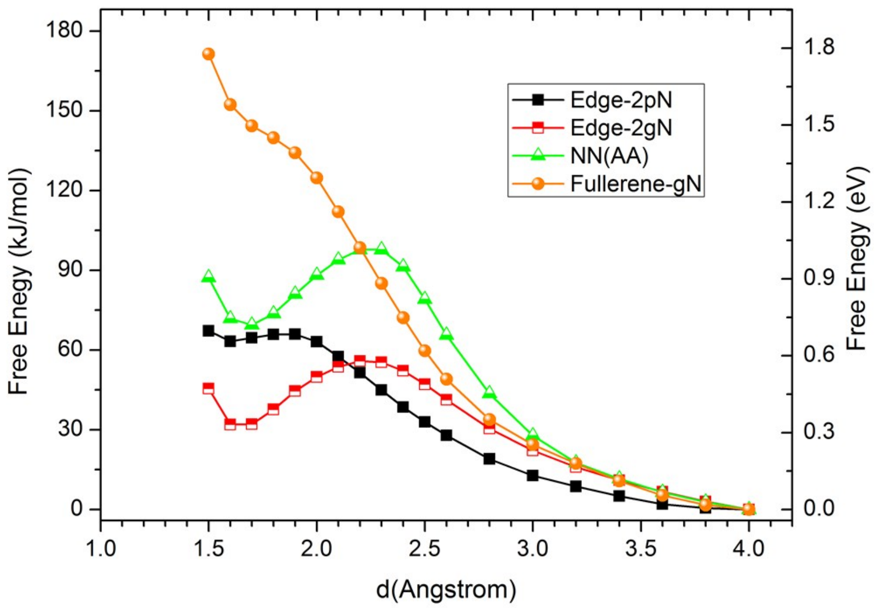

Figure S3: Free energy profiles of CO_2 approaching C sites labelled with a star as shown in Figure S4 for Edge-2pN, Edge-2gN, NN(AA) and Fullerene-gN. The approaching distance is the distance between C atom in CO_2 and the candidate site in the catalyst.

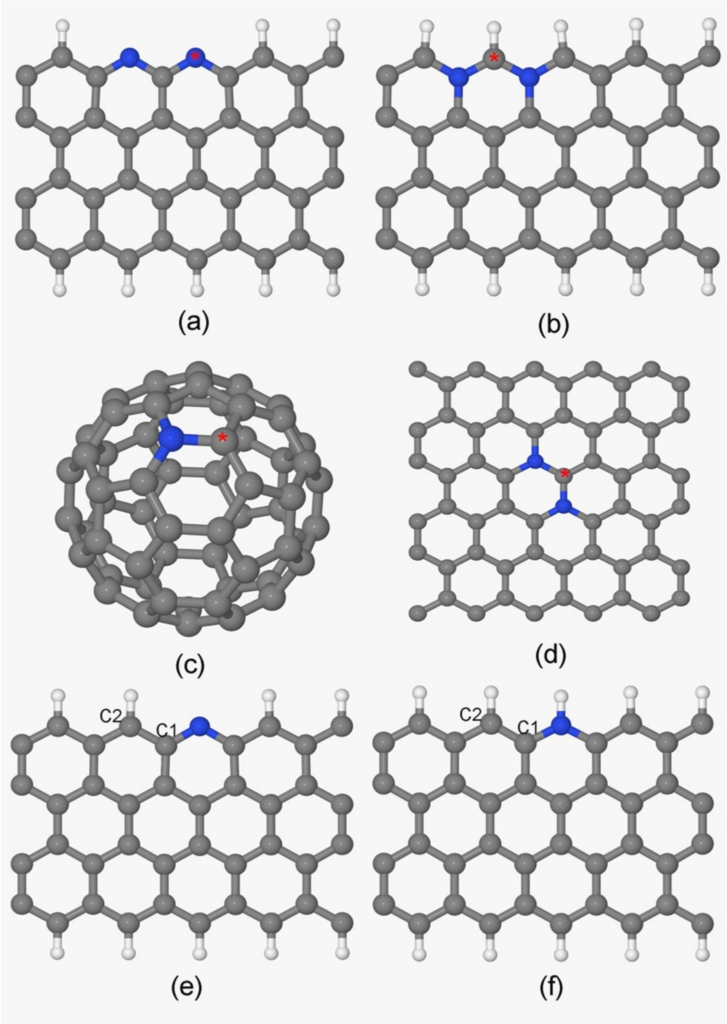

Figure S4: Unit cells for the periodic structures of (a) two pN doped zigzag edge Edge-2pN, (b) two gN doped zigzag edge Edge-2gN, (c) gN doped fullerene Fullerene-gN, (d) two gN doped in A sites of perfect graphene NN(AA), (e) one pN doped zigzag edge Edge-pN and (f) one pNH doped zigzag edge Edge-pNH, respectively. The grey, blue and white spheres represent carbon, nitrogen and hydrogen atoms, respectively.

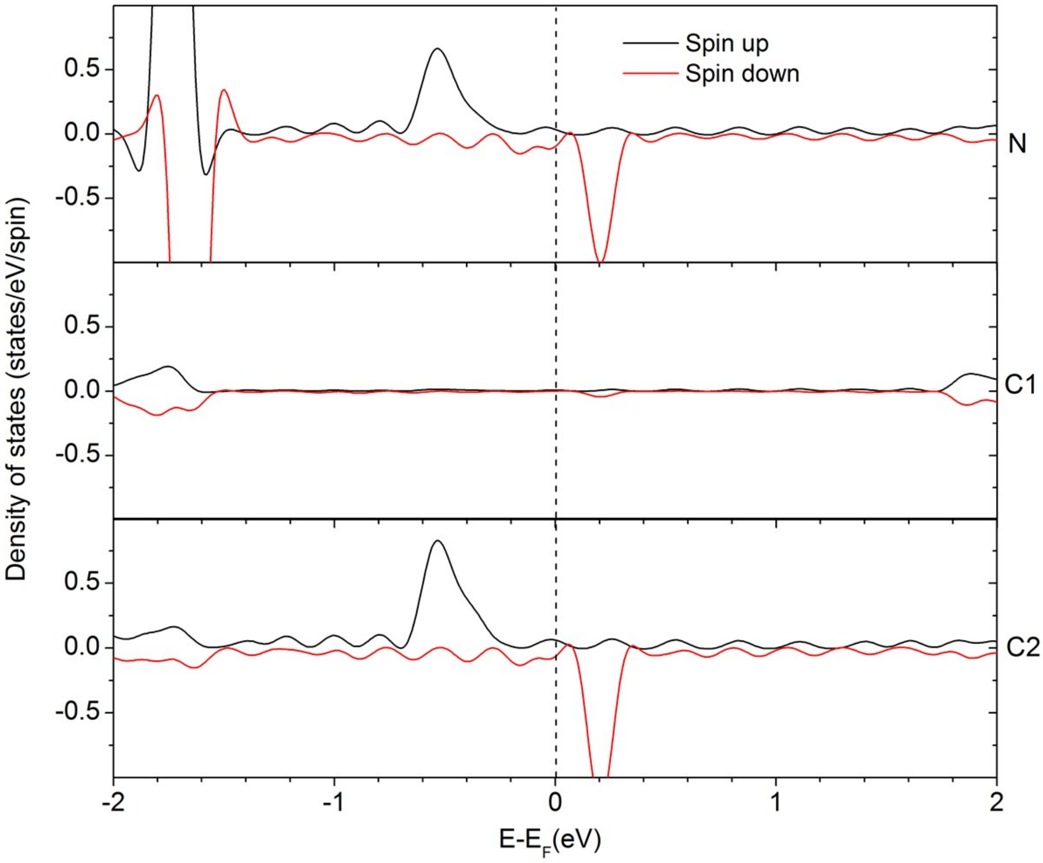

Figure S5: Density of states (DOSs) for Edge-pN structure. The corresponding structure is shown in Figure S4.

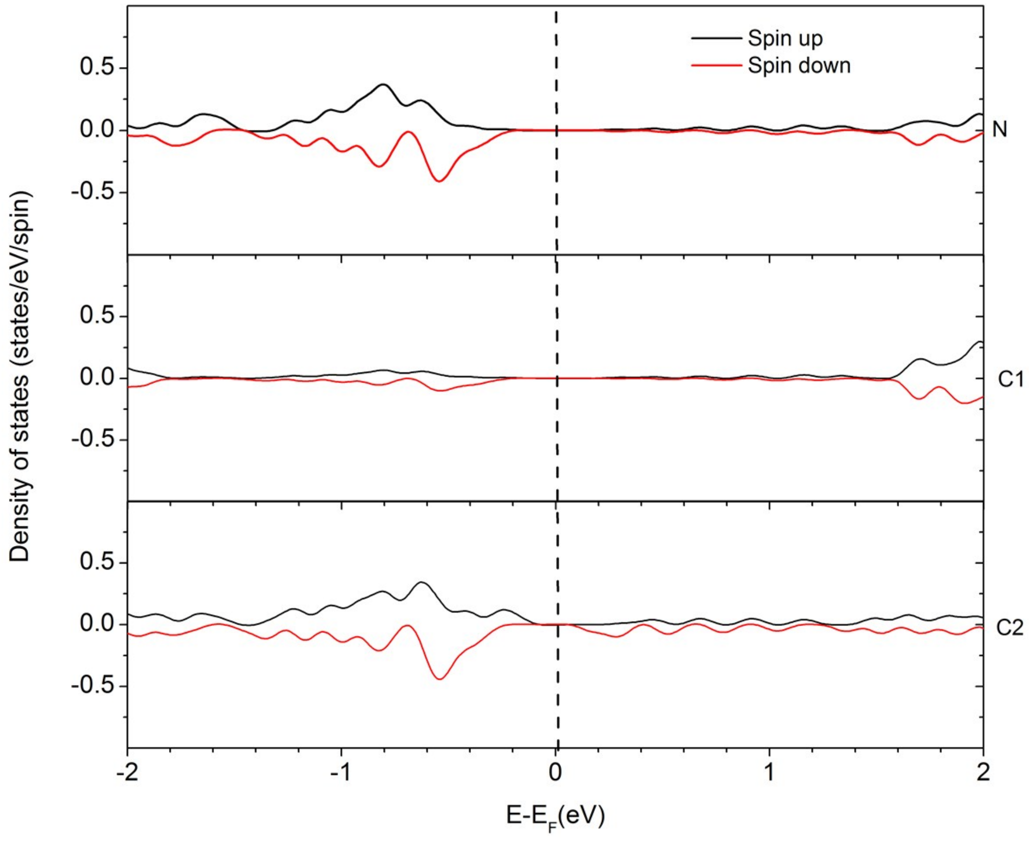

Figure S6: Density of states (DOSs) for Edge-pNH strucutre. The corresponding structure is shown in Figure S4.
